# Biomimetic Cell-Laden MeHA Hydrogels for the Regeneration of Cartilage Tissue

**DOI:** 10.3390/polym12071598

**Published:** 2020-07-18

**Authors:** Evgenia Tsanaktsidou, Olga Kammona, Norina Labude, Sabine Neuss, Melanie Krüger, Linda Kock, Costas Kiparissides

**Affiliations:** 1 Department of Chemical Engineering, Aristotle University of Thessaloniki, P.O. Box 472, 54124 Thessaloniki, Greece; jtsanaktsidou@certh.gr; 2 Chemical Process & Energy Resources Institute, Centre for Research and Technology Hellas, P.O. Box 60361, 57001 Thessaloniki, Greece; kammona@certh.gr; 3 Institute of Pathology, RWTH Aachen University Hospital, 52074 Aachen, Germany; nlabude@ukaachen.de (N.L.); sneuss-stein@ukaachen.de (S.N.); 4 Helmholtz-Institute for Biomedical Engineering, Biointerface Laboratory, RWTH Aachen University, 52074 Aachen, Germany; 5 LifeTec Group BV, 5611 ZS Eindhoven, The Netherlands; m.krueger@LifeTecGroup.com (M.K.); l.kock@lifetecgroup.com (L.K.)

**Keywords:** methacrylated hyaluronic acid, injectable cell-laden HA hydrogels, matrix metalloproteinase-degradable peptide, chondroitin sulfate-binding peptide, human mesenchymal stem cells, chondrocytes, cartilage repair, ex vivo model

## Abstract

Methacrylated hyaluronic acid (MeHA) and chondroitin sulfate (CS)-biofunctionalized MeHA (CS-MeHA), were crosslinked in the presence of a matrix metalloproteinase 7 (MMP7)-sensitive peptide. The synthesized hydrogels were embedded with either human mesenchymal stem cells (hMSCs) or chondrocytes, at low concentrations, and subsequently cultured in a stem cell medium (SCM) or chondrogenic induction medium (CiM). The pivotal role of the synthesized hydrogels in promoting the expression of cartilage-related genes and the formation of neocartilage tissue despite the low concentration of encapsulated cells was assessed. It was found that hMSC-laden MeHA hydrogels cultured in an expansion medium exhibited a significant increase in the expression of chondrogenic markers compared to hMSCs cultured on a tissue culture polystyrene plate (TCPS). This favorable outcome was further enhanced for hMSC-laden CS-MeHA hydrogels, indicating the positive effect of the glycosaminoglycan binding peptide on the differentiation of hMSCs towards a chondrogenic phenotype. However, it was shown that an induction medium is necessary to achieve full span chondrogenesis. Finally, the histological analysis of chondrocyte-laden MeHA hydrogels cultured on an ex vivo osteochondral platform revealed the deposition of glycosaminoglycans (GAGs) and the arrangement of chondrocyte clusters in isogenous groups, which is characteristic of hyaline cartilage morphology.

## 1. Introduction

Glass-like, avascular and aneural articular cartilage tissue, lining the ends of articulating bones, is characterized for its minimal friction and ability to tolerate heavy loads, thus facilitating the movement of one bone against another. Articular cartilage consists of water (65–80 wt%) and solids comprising mainly collagens (50–75 dry wt%) and proteoglycans (15–30 dry wt%), as well as protein molecules and chondrocytes [1,2]. The solids form a three-dimensional network responsible for the good mechanical properties of articular cartilage. As a result of cartilage avascularity, low chondrocyte proliferative activity, and high extracellular matrix protein density, the intrinsic healing ability of the native cartilage tissue is limited and thus no tissue regeneration can be observed following cartilage injuries like chondral defects, microfractures, etc. Two common operative procedures (i.e., the autologous chondrocyte implantation, ACI, and the matrix-associated autologous chondrocyte implantation, MACI) used for cartilage repair exhibit limitations with regard to the cartilage defect size and geometry [1,3,4,5,6,7]. On the other hand, non-invasive topical administration via the injection of in situ forming cell-laden hydrogels could offer a promising alternative to the above operative procedures [8].

Hydrogels are three-dimensional networks of physically or chemically crosslinked hydrophilic, natural, or synthetic polymer chains. These networks can be easily formed under mild reaction conditions and tuned with regard to their molecular (i.e., degree of crosslinking and swelling), viscoelastic (i.e., storage and loss moduli), and mechanical (i.e., compressive modulus) properties and degradation kinetics [9,10,11]. They can also be functionalized with appropriate peptides to increase cell adhesion to the hydrogel matrix [10]. In addition, cell-laden hydrogels form a protective environment that mimics the native tissue, allows the diffusion of solutes (e.g., growth factors and cell signaling molecules), and promotes the growth of new tissue [8]. Finally, in situ gelation following topical injection permits the hydrogel to accurately and completely fill in irregularly shaped cartilage defects [12]. 

Hyaluronic acid (HA), is a natural glycosaminoglycan (GAG) present in the extracellular matrix of connective tissues, consisting of repeating disaccharide units (i.e., D-glucuronic acid and N-acetyl-D-glucosamine) [11,12]. Following the chemical modification of HA with appropriate functional groups (e.g., methacrylates [13]), HA-based hydrogels can be typically formed via covalent crosslinking. They are biocompatible and biodegradable and susceptible to enzymatic degradation but not to hydrolysis. Moreover, HA hydrogels favor the differentiation of human mesenchymal stem cells (hMSCs) towards a chondrogenic phenotype and, consequently, the formation of neocartilage tissue [12,14]. Note that the chondrogenesis properties of HA hydrogels can be further enhanced by the functionalization of HA hydrogels with adhesion peptides (e.g, glycosaminoglycan-binding peptides, arginyl-glycyl-aspartic acid (RGD), etc.) [12,13,15,16]. Thus, HA-based hydrogels are considered to be an excellent choice as cell carriers for cartilage repair [17].

Hydrogel degradation, affecting the diffusion of solutes, hydrogel–cell interactions, and the distribution of extracellular matrix (ECM) proteins, should preferably match the deposition and accumulation of ECM. Cell-mediated hydrogel degradation is effected by the action of catabolic enzymes (e.g., matrix metalloproteinases, MMPs) secreted by the cells [18]. More specifically, matrix metalloproteinase 7 has been identified as the only catabolic enzyme that exhibits both a low expression in hMSCs and a relatively increasing expression during the in vitro culture of hMSCs in chondrogenic induction medium CiM [19]. Accordingly, hydrogel degradation has been tailored via the use of MMP-degradable peptide crosslinkers [20,21,22,23,24,25]. In particular, MMP7-degradable peptides have been successfully used as crosslinkers for the formation of polyethylene glycol (PEG) [19,26], and methacrylated hyaluronic acid (MeHA) [13] based hydrogels, as well as hydrogels based on a recombinant streptococcal collagen-like 2 (Scl2) protein [15,27,28]. 

Chondrocytes are the most apparent choice for the cell seeding of hydrogels since they are found in native cartilage and their role is to maintain/remodel the surrounding ECM [29]. It has been shown that articular chondrocytes, and in particular freshly isolated ones [30], are able to proliferate in hydrogels, exhibit appropriate cell morphology and phenotype, express cartilage-related genes/proteins [31], and produce neocartilage tissue with superior mechanical properties [32]. However, despite the promising results in cartilage tissue engineering (TE), inherent limitations regarding the collection of healthy cartilage tissue for chondrocyte harvesting, long-term in vitro cell culturing [31], low cell yields, and tendency to cell de-differentiation [33] during in vitro expansion, have motivated the use of MSCs, which can be effortlessly expanded in vitro without losing their differentiation potential [21]. MSCs have been extensively used in TE applications because of their low immunogenicity, abundance of cell sources, etc. [31]. Among the different MSC sources, bone marrow-derived MSCs have been found to exhibit the greatest chondrogenic potential [34,35]. Note that increased collagen deposition has been observed in TE constructs seeded with either chondrocytes or MSCs at various seeding densities [36].

In the present study, MeHA hydrogels were synthesized using an MMP7-degradable peptide and functionalized with a chondroitin sulfate (CS)-binding peptide [13]. Accordingly, the MeHA-based hydrogels were loaded with hMSCs and thoroughly assessed with respect to their ability to enhance the proliferation and growth of hMSCs and favor the differentiation of hMSCs to chondrocytes while suppressing their differentiation rate towards a hypertrophic phenotype. Moreover, chondrocyte-laden MeHA hydrogels were assessed regarding their compatibility with chondrocytes and ability to form neocartilage tissue using an ex vivo model (i.e., osteochondral culture platform).

## 2. Materials and Methods 

### 2.1. Materials

Hyaluronic acid (MW: 66–99 kDa) was purchased from Lifecore (Chaska, MN, USA). Recombinant human matrix metalloproteinase-7 (MMP7) from a mouse myeloma cell line (specific activity > 600 pmol/min/μg, endotoxin level less than 1.0 EU per 1 μg of protein) was purchased from RnD Systems, Minneapolis MN, USA. Methacrylic anhydride (MA), N,N-dimethylformamide (for molecular biology ≥99%), triethanolamine (≥99.0%, GC), ethylenediaminetetraacetic acid (ACS reagent, 99.4–100.6%, powder), magnesium sulfate anhydrous, sodium chloride (ACS reagent, ≥99.0%), fluoresceindiacetate, propidium iodide, and papain from papaya latex were purchased from Merck (Darmstadt, Germany).. 

### 2.2. Synthesis and Functionalization of MeHA

**Figure 1:**
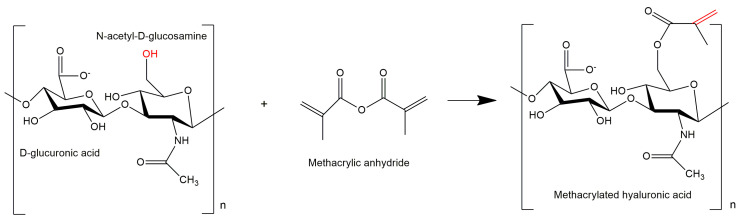
Modification of hyaluronic acid using methacrylic anhydride.

The synthesis of methacrylated hyaluronic acid (MeHA) and its functionalization with a chondroitin sulfate (CS)-binding peptide have been described in detail in our previous publication [13]. In brief, HA (MW: 66–99 kDa) was modified via its reaction with methacrylic anhydride (MA) (Figure 1) in a water–N,N-dimethylformamide (DMF) mixture (1/1 v/v). In particular, 0.095 g or 0.25 mmol of HA were dissolved in 10 mL of ultrapure water. DMF was subsequently added dropwise to the aqueous solution of HA until a final water/DMF ratio of 1/1 v/v. MA (0.385 g or 2.5 mmol) was then added to the polymer solution and the pH of the reaction medium was adjusted to a value of 8–9. The reaction was carried out at a temperature of 4 °C for 24 h under the aid of a magnetic stirrer. The synthesized MeHA was purified via successive centrifugation/washing cycles and dialysis against water (MWCO 7 kDa), and was finally recovered by lyophilization [13]. The degree of methacrylation (DM) was determined by ^1^H NMR spectroscopy (Varian 600 MHz spectrometer, Agilent, Santa Clara, CA, USA). Briefly, MeHA was dissolved in deuterated water (D_2_O) at 25 °C and ^1^H NMR spectra were recorded and subsequently processed using Mnova software [13,37,38].

In a subsequent step, MeHA was functionalized with a CS-binding peptide (CGGGYKTNFRRYYRF) via a thiol-methacrylate chemical reaction. Briefly, 0.02 g of MeHA were dissolved in ultrapure water, containing 0.2 M triethanolamine (TEA), under magnetic stirring at 25 °C. Then, 0.00087 g or 0.00046 mmol of CS-binding peptide (stoichiometrically corresponding to 1% of the methacrylates in MeHA) were dissolved in ultrapure water containing 0.2 M TEA. The CS solution was then added to the MeHA solution. The reaction of MeHA with CS was carried out, under magnetic stirring, at 37 °C for 24 h. The synthesized CS-MeHA was purified via dialysis against water (MWCO 7 kDa) and was finally recovered by lyophilization. The functionalization of MeHA with the CS-binding peptide was verified by ^1^H NMR spectroscopy [13].

### 2.3. Formation of MeHA and CS-MeHA Hydrogels

MeHA and CS-MeHA hydrogels were formed via the crosslinking of MeHA or CS-MeHA with the MMP7-degradable peptide (CGGGPLELRAGGGC). Accordingly, a specific quantity of MeHA or CS-MeHA (16 mg) was first dissolved in 400 μL of Dulbecco’s modified Eagle’s medium (DMEM; Gibco) containing 0.1 M TEA at pH 8.0. A predetermined amount (0.0072 mmol) of the MMP7-degradable peptide, stoichiometrically corresponding to 40% of the methacrylates in the macromer (i.e., MeHA or CS-MeHA) was dissolved in 100 μL of ultrapure water containing 0.1 M TEA. Then, the two solutions were thoroughly mixed at 37 °C, followed by the crosslinking of HA polymer chains for approximately 8 min. The progress of the crosslinking reaction was monitored via the measurement of storage, G’, and loss, G’’, moduli with the aid of a cone and plate dynamic stress rheometer (SR-5000, Rheometrics Scientific, Piscataway, NJ, USA) at 37 °C, operating at 1% strain and a frequency of 1 rad/s [13]. The degree of swelling of the synthesized MeHA and CS-MeHA hydrogels was measured by the standard gravimetric method. Consequently, hydrogel discs of 10 mm in diameter and 2 mm in width, prepared using custom-made Teflon molds, were first dried, weighed, and incubated in PBS (at 37 °C). At specified times, the discs were removed from the PBS, wiped with filter paper, and weighed. The degree of hydrogel swelling was calculated by the following equation [39]:

Degree of swelling (%) = [(W_s_ − W_d_)/W_d_] × 100 (1)

where W_s_ and W_d_ are the weights of the hydrogel in the swollen and dry state, respectively.

The degradation kinetics of MeHA and CS-MeHA hydrogels were experimentally studied by measuring the weight loss (%) of hydrogel discs with respect to time. In this direction, MeHA and CS-MeHA hydrogel discs were first swollen in PBS, at 37 °C for 3 days, and then placed in 2 mL degradation medium containing 30 ng/mL of recombinant human MMP7 in PBS. The degradation medium was refreshed every 2 days during the kinetic experiment to ensure a sustained enzyme activity. At specified time intervals, hydrogels were removed from the MMP7 solution, wiped with filter paper, and weighed. Thus, the weight loss of degrading MeHA and CS-MeHA hydrogels could be measured with time [13].

### 2.4. In Vitro Assessment of hMSC-Laden MeHA and CS-MeHA Hydrogels

#### 2.4.1. Mesenchymal Stem Cell Culture

Human mesenchymal stem cells (hMSCs) were isolated from the femoral heads of three donors. The femoral heads were extracted during hip joint surgery with patients’ consent, at the university hospital of Aachen, Germany (Ethics commitee vote EK 300/13) [40]. The isolated hMSCs were seeded in a T75 flask containing a stem cell culture medium (SCM) in a CO_2_ incubator (37 °C, 5% CO_2_). The medium was changed twice per week and, at cell a confluency percentage of around 80–90%, hMSCs were trypsinized with 2.5% trypsin-EDTA, centrifuged, and subcultured in SCM. The hMSCs expanded to passage 2 were subsequently used for cell encapsulation studies [15].

#### 2.4.2. Cell Encapsulation

A specific amount of MeHA or CS-MeHA (16 mg) was dissolved in Dulbecco’s modified Eagle’s medium (DMEM; Gibco, Thermo Fisher, Paisley, UK), containing 0.1 M TEA at pH = 8.0. The hMSCs, at a density of 1 × 10^6^ cells/mL, were homogeneously dispersed in the polymer solution. Subsequently, the cell-containing polymer solution was mixed with an MMP7-degradable peptide solution in ultrapure water containing 0.1 M TEA. Aliquots of 50 μL of the resulting mixture (5 w/v% solid content) were placed in a non-treated 48-well plate and allowed to gel for 1 h in a CO_2_ incubator (37 °C, 5% CO_2_). The cell-laden hydrogels were then cultured in 500 μL of SCM (Mesenpan, PAN biotech, Aidenbach, Germany), consisting of 60% DMEM low glucose, 40% MCDB-201, 1x insulin-transferrin-selenium (ITS) with BSA-linoleic acid, 1 nM dexamethasone, 100 μM ascorbic acid 2- phosphate, 40,000 units penicillin, 40 mg/mL streptomycin, 10 ng/mL epidermal growth factor (EGF), and 2 wt% fetal calf serum (FCS). Alternatively, hydrogels were cultured in 500 μL of CiM (StemProChondorgenesis differentiation Kit; Gibco, Thermo Fisher, Paisley, UK). The cell-laden hydrogels were cultured for 7 and 14 days, while the medium was changed three times a week. The hMSCs were also cultured for 7 and 14 days on tissue culture polystyrene (TCPS) in SCM or CiM as a control.

#### 2.4.3. Cell Viability

The hMSC-laden hydrogels were assessed for cell viability with a live/dead staining assay according to the 10993-5 protocol of the International Standardization Organization (ISO) [41]. After 7 and 14 days of cell culture, the culture medium was removed and the cell-laden hydrogels were stained with propidium iodide (indicator of dead cells) and fluorescein diacetate (indicator of viable cells) dissolved in Ringer’s solution (FDA: 1.6 v/v% and PI: 1.6 v/v%). The hydrogels were incubated with the staining solution for a few seconds at room temperature before cell visualization at different z-dimensions with the aid of a fluorescence microscope (Microscope Leica DMI6000 B Fluorescence, Leica, Wetzlar, Germany). The percentage cell viability was calculated using ImageJ software, based on measurements of live and dead cells from five different regions of the MeHA hydrogels.

#### 2.4.4. Biochemical Analysis

Following a culture period of 7 and 14 days, hMSC-laden hydrogels were washed three times with PBS and then digested in a papain digestion solution (2.5 U/mL papain, 5 mM cysteine HCl, 5 mM EDTA, in PBS) for 16 h at 60 °C in order to determine the DNA content. More specifically, the Quant-iT™ PicoGreen^®^ dsDNA assay kit (Invitrogen) was used to determine the DNA content using Lambda phage DNA (0–25 ng/mL) as a standard. The assay was performed according to the manufacturers’ instructions and the measurements were carried out in a fluorescence microplate reader (NanoDrop 1000, Fisher scientific, Waltham, MA, USA) [14,15,42]. 

#### 2.4.5. Gene Expression Analysis

For gene expression analysis, hMSC-laden hydrogels cultured for 7 and 14 days were homogenized in Trizol reagent (Invitrogen) using a tissue raptor [14,15]. Total RNA was isolated using the NucleoSpin RNA XS Kit (Macherey-Nagel, Fisher Scientific, Loughborough, UK) and reverse transcribed into cDNA with the High-Capacity cDNA Reverse Transcriptase Kit (Applied Biosystems, Foster City, CA, USA). Both RNA and cDNA concentrations were spectrophotometrically assayed (NanoDrop 1000 spectrophotometer, Fisher Scientific, Waltham, MA, USA)). Real-time polymerase chain reaction (real-time PCR) was performed for cDNA samples of 1.2 μL, using the Power SYBR Green Master Mix (ThermoFisher, Waltham, MA, USA) and the 7300 Realtime PCR system (Applied Biosystems, Foster City, CA, USA). The genes of interest were normalized to the reference gene, GAPDH, and the data were analyzed using the 2^(−ΔΔCT)^ method. Table 1 shows the sequences of the primers that were used [14,15,40].

### 2.5. Ex Vivo Assessment of Chondrocyte-Laden MeHA Hydrogels

#### 2.5.1. Isolation of Porcine Chondrocytes

Chondrocytes were isolated from the cartilage of porcine knee joints that were provided by a slaughterhouse in Boxtel, the Netherlands. The knee joints were from Dutch Land Raise hybrid pigs, male or female, 5–8 months old, and 100–110 kg live weight [43]. According to a protocol provided by LifeTec Group B.V., small pieces of cartilage were removed from the joint, under sterile conditions, taking care not to remove the calcified tissue. Two grams of cartilage pieces were subsequently digested in 15 mL of 0.5 v/v% collagenase II (Worthington) in basic bone medium (high glucose DMEM, 10 v/v% FBS, 2 v/v% pen/strep, and 2 v/v% amphotericin B). After the digestion procedure was completed, the cells were filtered three times with a cell strainer and flushed with basic bone medium. Finally, the total cell number was determined and the isolated chondrocytes were stored in cartilage medium (HG DMEM, 1 v/v% ITSTM Premix, 50 μg/mL L-ascorbic acid–2-phosphate, 40 μg/mL L-proline, 1 mM sodium pyruvate, 2.5 μg/mL amphotericin B, 100 U/mL penicillin/streptomycin) at 37 °C [43].

#### 2.5.2. Ex Vivo Explant Study

**Figure 2:**
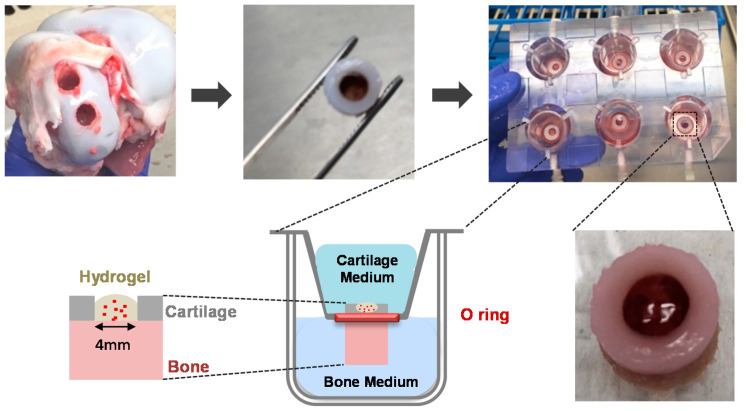
Ex vivo osteochondral platform: isolation of osteochondral explants; formation of cartilage defects; mounting of the osteochondral explants; filling of osteochondral explants with chondrocyte-laden methacrylated hyaluronic acid MeHA hydrogels.

The ability of MeHA chondrocyte-laden hydrogels to induce cartilage lesion repair was assessed in an ex vivo cartilage model (LifeTec Group B.V.). Osteochondral explants were initially drilled from the medial side of the femoral condyle of knee joints of 5–7-month-old, male or female, Dutch landrace hybrid pigs, with a dental drill bit (Ø 0.8 cm, Makita drill, DDF343, 14.4 V, RS Components Ltd, Corby, UK), while being cooled with sterile PBS containing 2 v/v% penicillin/streptomycin and 2 v/v% amphotericin B. A custom-made removal tool was placed around the drilled explants and the plug was removed from the joint by applying pressure to all sides of the removal tool. A sawing procedure followed the drilling of the osteochondral explants. The bone of the plug was sawn to a specific length of 4 mm, which could perfectly fit a custom-made osteochondral platform. A biopsy tool (PMF Medical) of 4 mm in diameter, was then used to punch a hole in the center of the explants and a sharp spoon (MF Dental) was used to remove the remaining cartilage [43,44], thus creating a chondral defect to be subsequently filled with the cell-laden hydrogel. The defect-bearing plugs were subsequently mounted onto the osteochondral platform. More specifically, each explant was placed in an insert containing an O-Ring of 8 mm in diameter, which was positioned at the interface between the cartilage and the bone. The insert was then suspended in a custom-made six-well plate. In this way, two separate compartments were created, i.e., an upper compartment for the defected cartilage and a lower compartment for the bone (Figure 2). 

MeHA (16 mg) was dissolved in Dulbecco’s modified Eagle’s medium (DMEM; Gibco, Thermo Fisher, Paisley, UK) containing 0.1 M TEA at pH = 8.0. Isolated porcine chondrocytes were homogeneously dispersed in the MeHA solution at a density of 1.25 × 10^6^ cells/mL. Subsequently, the cell-containing macromer solution was mixed with an MMP7-degradable peptide solution in ultrapure water containing 0.1 M TEA. Aliquots of 40 μL of the resulting mixture (hydrogel content 5 and 7.5 w/v%) were placed in the defects of the osteochondral explants and allowed to gel for 1 h in a CO_2_ incubator (37 °C, 5% CO_2_), as shown in Figure 2. Subsequently, the upper compartment, containing the defected cartilage, was filled with 3 mL of cartilage medium, whereas the lower compartment, containing the bone, was filled with 3 mL of bone medium (DMEM HG, 2.5 μg/mL amphotericin B, 100 U/mL penicillin/streptomycin, 100 nM dexamethasone, 10 v/v% FBS, 10 mM B-glycerophosphate, 50 μg/mL L-ascorbic acid-2-phosphate) [43,44]. The chondrocyte-laden MeHA hydrogels were cultured in the ex vivo model for 7 and 14 days and the cartilage and bone media were changed twice per week.

#### 2.5.3. Metabolic Activity of Chondrocytes

The metabolic activity of the encapsulated chondrocytes was assessed in vitro, on days 1, 7, and 14 of culture, with the PrestoBlue cell viability assay (A-13261 Invitrogen) according to the manufacturer’s instructions. Initially, the chondrocyte-laden MeHA hydrogels were removed from the osteochondral platform and placed on tissue culture polystyrene (TCPS) with 90 μL basic cartilage medium and 10 μL PrestoBlue reagent. The TCPS 96-well plate was protected from light and placed in a CO_2_ incubator (37 °C, 5% CO_2_) for 3.5 h. Subsequently, the plate was shaken for 30 min by means of an orbital shaker. Finally, the dye solution (100 μL) was transferred to a black 96-well plate suitable for fluorometric assays and the fluorescence was read out with a plate reader (CLARIOstar microplate reader, BMG Labtech, Ortenberg, Germany) at an excitation wavelength of 535 nm and an emission wavelength of 615 nm [43].

#### 2.5.4. Histological Analysis

Histological analysis was performed for chondrocyte-laden MeHA hydrogels cultured for 7 and 14 days, following their extraction from the explants. In detail, MeHA hydrogels containing chondrocytes were fixed in 3.7% formalin (Sigma, Zwijndrecht, The Netherlands) for 1 h at room temperature and then transferred to a 70% ethanol solution where they remained overnight at 4 °C. Following the removal of the infiltration solution, hydrogels were placed in cryomolds filled with OCT (Tissue-Tek, Sakura) and were immersed in isopentane to freeze (Snapfrost^®^2, Excilone, Elancourt, France) before being cut in the cryostat (MNT, SLEE medical, Mainz, Germany). Frozen sections 5 μm thick were thus obtained and subsequently placed on polysine slides. 

To examine the distribution of the encapsulated chondrocytes, the above-mentioned frozen hydrogel sections were stained with hematoxylin and eosin (H&E). Additionally, Safranin O/Fast Green staining was applied to the hydrogel samples in order to examine the accumulation of GAGs produced by the encapsulated chondrocytes and the formation of ECM. Both methods were performed according to standard protocols.

### 2.6. Statistical Analysis

The results are presented as mean values ± standard error of the mean (SEM). The gene expression data were analyzed employing a two-way analysis of variance (ANOVA), using (i) the type of culture medium and the culture period, (ii) the hydrogel type and the culture period, and iii) the hydrogel type and the type of culture medium, as variables. One-way ANOVA was used for the DNA quantification and one-way ANOVA with Tukey’s post hoc analysis was used for the metabolic activity assay. SPSS software was applied for the statistical analysis. The results were significant for *p*-values < 0.05.

## 3. Results

### 3.1. Formation of MeHA and CS-MeHA Hydrogels

MeHA with a degree of methacrylation (DM%) equal to 46.5 ± 5.5 [13] and CS-MeHA with a theoretical degree of biofunctionalization equal to 1% [13] were used in the synthesis of cell-laden MeHA and CS-MeHA hydrogels, respectively.

**Figure 3:**
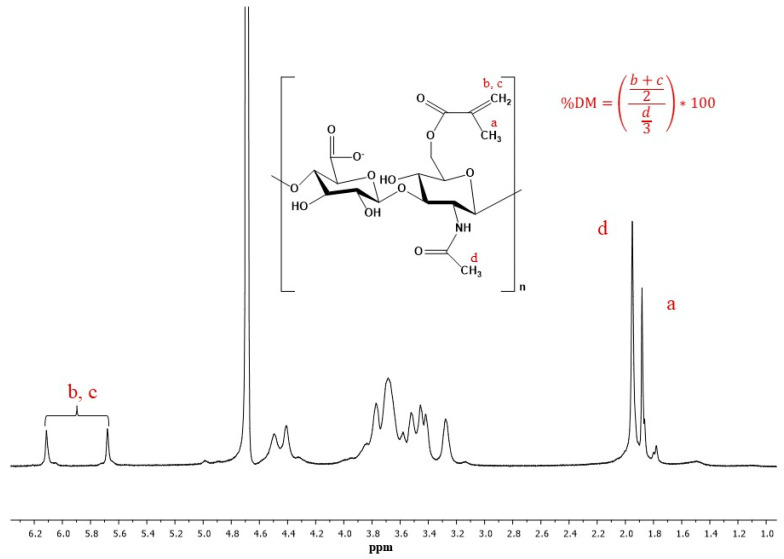
^1^H NMR spectrum of MeHA.

Figure 3 shows an indicative ^1^H NMR spectrum of MeHA, where the characteristic peaks of MeHA at δ 6.1, 5.7 corresponding to the methacrylate CH_2_ group, indicating the methacrylation of HA, can be clearly distinguished. In this case, DM is equal to 47.06% as calculated by the ratio of the methacrylate proton peaks at 6.1 and 5.7 to the peak at 1.9 corresponding to the CH_3_ of the N-acetyl glucosamine of HA [13,37,38].

In Table 2, the gelation onset time and storage modulus (G’) of MeHA and CS-MeHA hydrogels synthesized in the presence of an MMP7-degradable peptide are reported. It is apparent that CS-MeHA hydrogels exhibit an increased gelation onset time and a decreased G’ value compared with those of MeHA hydrogels. 

**Figure 4:**
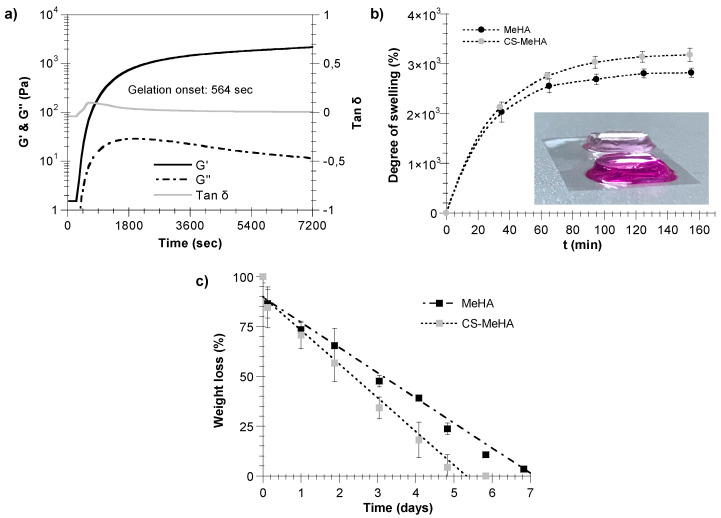
(**a**) Monitoring of gelation kinetics of CS-MeHA in the presence of an MMP7-degradable crosslinker. Time variation of storage, G’, and loss modulus, G”. CS-MeHA characteristics (MW of HA: 66–99 kDa, DM of MeHA: 48.8%, theoretical degree of biofunctionalization of MeHA: 1%, CS-MeHA hydrogel solids: 5 w/v%, moles of MMP7-degradable peptide: 0.0072 mmol). (**b**) Swelling rate of MeHA and CS-MeHA hydrogels in PBS. Results are presented as mean values of three experiments. Error bars show ± standard error of the mean. Insert: MeHA (light pink) and CS-MeHA (magenta) hydrogels swollen with PBS. (**c**) Degradation kinetics of MeHA and CS-MeHA hydrogels in a recombinant human MMP7 solution in PBS (30 ng/mL) at 37 °C. Results are presented as mean values of three experiments. Error bars show ± standard error of the mean.

In Figure 4a, the time evolution of the storage, G’, and loss, G’’, moduli of the CS-MeHA hydrogel are illustrated. For this system, the gelation onset time, determined by the change in the slope of tanδ, was equal to 564 s, whereas the storage modulus increased to a value of 2180 Pa. Note that the measured gelation onset time of the CS-MeHA hydrogel should be larger than the administration time of the injectable formulation (i.e., the solution of macromer and crosslinker), as well as greater than the spreading time of the reactive solution for the complete coverage of the cartilage defect. 

Figure 4b illustrates the degree of swelling of the synthesized MeHA and CS-MeHA hydrogels with respect to time. As can be seen, the uptake of PBS increases with time until it reaches an equilibrium swelling value, directly related to the degree of hydrogel crosslinking. Actually, the equilibrium swelling value increases as the degree of hydrogel crosslinking decreases (e.g., CS-MeHA hydrogels). Note that the results of Figure 4b are in agreement with the respective values of the storage modulus for the MeHA and CS-MeHA hydrogels shown in Table 2.

Figure 4c depicts the degradation kinetics of MeHA and CS-MeHA hydrogels in a recombinant human MMP7 solution in PBS (30 ng/mL) at 37 °C. As can be seen, in both cases, the hydrogel degradation exhibits a zero-order kinetic model (i.e., the hydrogel weight loss percentage with respect to time follows a straight line). It is evident that the CS-MeHA hydrogel exhibits a higher degradation rate than that of MeHA hydrogel.

### 3.2. In Vitro Assessment of hMSC-Laden MeHA and CS-MeHA Hydrogels

#### 3.2.1. Cell Viability

**Figure 5:**
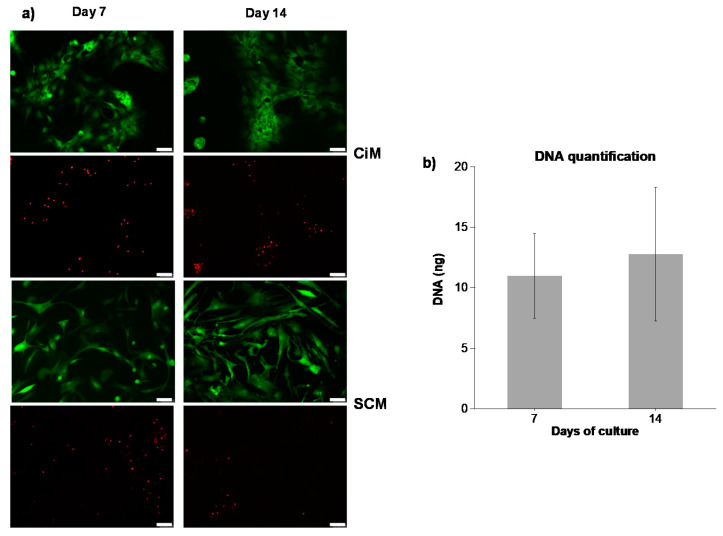
(**a**) Live/dead staining of human mesenchymal stem cells (hMSCs) encapsulated in MeHA hydrogels (5 w/v%) and cultured in chondrogenic medium (CiM) or stem cell medium (SCM) after 7 and 14 days of culture. Scale bar = 75 μm. (**b**) DNA amount of hMSC-laden MeHA hydrogels cultured in SCM for 7 and 14 days.

Figure 5a illustrates the live/dead staining of hMSCs encapsulated in MeHA hydrogels (5 w/v%) and cultured in two different media, namely (i) SCM (expansion medium) and (ii) CiM (induction medium). As can be seen, the viability of hMSCs does not change during the culture period independently of the culture medium used. It should be noted that following 14 days of culture, approximately 80% of the incorporated cells remained viable. Regarding cell morphology, hMSCs cultured in SCM appear to maintain their spindle-like cell shape, with a small number of cells having a round morphology. On the other hand, those cultured in CiM exhibit a chondrocyte-like, round shape and form an interconnected network, indicating the differentiation of hMSCs towards a chondrogenic phenotype.

#### 3.2.2. Biochemical Analysis

Figure 5b shows the amount of DNA in hMSC-laden MeHA hydrogels cultured in SCM following 7 and 14 days of culture. As can be seen, the DNA content does not change significantly between days 7 and 14 of cell culture. Nevertheless, it exhibits an increasing trend. This observation is in agreement with the cell viability results presented in Figure 5a for cell-laden hydrogels cultured in SCM, which show that hMSCs grow and proliferate throughout the culture period.

#### 3.2.3. Gene Expression Analysis

**Figure 6:**
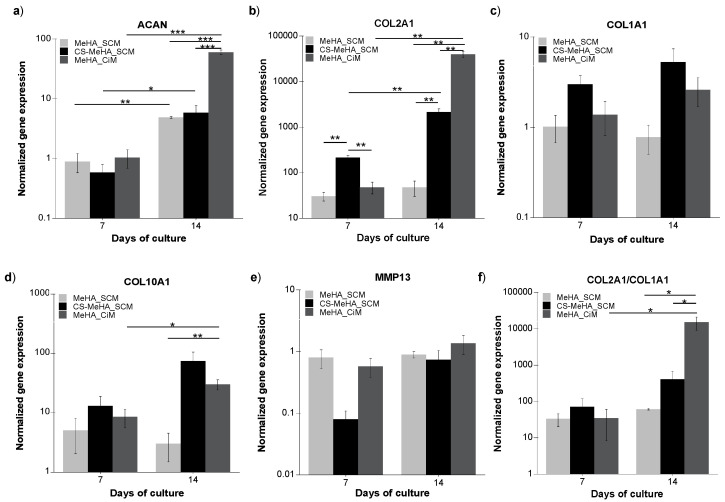
Gene expression analysis of (**a**) ACAN, (**b**) Col2A1, (**c**) Col1A1, (**d**) Col10A1, and (**e**) MMP13 for hMSCs encapsulated in MeHA hydrogels cultured in SCM or CiM and CS-MeHA hydrogels cultured in SCM, following 7 and 14 days of in vitro culture. (**f**) Col2A1/Col1A1 ratio of gene expression. The data were normalized to GAPDH and expressed as fold difference relative to undifferentiated hMSCs cultured on a TCPS. The results were analyzed using two-way analysis of variance (ANOVA) and are presented as mean values ± standard error of the mean, * *p* < 0.05, ** *p* < 0.01, *** *p* < 0.001 (*n* = 3 for each donor, hMSCs derived from three different donors).

Figure 6 depicts the gene expression of the chondrogenic markers ACAN (6a) and Col2A1 (6b), and the hypertrophic markers Col1A1 (6c), Col10A1 (6d), and MMP13 (6e) following 7 and 14 days of in vitro culture of hMSC-laden MeHA hydrogels in SCM and CiM, and CS-MeHA hydrogels in SCM.

Regarding the comparison of hMSC-laden MeHA hydrogels cultured in different media, it is apparent that there are no significant differences in the gene expression of both chondrogenic and hypertrophic markers following 7 days of culture in SCM or CiM (Figure 6a–e). Additionally, no significant differences can be seen in the expression of Col1A1 and MMP13 following 14 days of culture in both media (Figure 6c,e). On the other hand, the expression of the chondrogenic markers Col2A1 and ACAN, as well as of the hypertrophy marker Col10A1, is significantly higher on day 14 of culture for hydrogels cultured in CiM compared to hMSC-laden MeHA hydrogels cultured in SCM (Figure 6a,b,d). Regarding the effect of culture period, significant differences can be seen in the expression of Col2A1, ACAN, and Col10A1 between days 7 and 14 for hMSC-laden hydrogels cultured in CiM (Figure 6a,b,d), whereas for those cultured in SCM, significant differences can only be observed in the expression of ACAN (Figure 6a). Concerning the variation of the Col2A1/Col1A1 ratio, a cell differentiation index [15], one can observe a significant increase from day 7 to day 14 of culture for hMSC-laden MeHA hydrogels cultured in CiM (Figure 6f). Furthermore, as can be seen in Figure 6f, the Col2A1/Col1A1 ratio for hMSC-laden hydrogels cultured in CiM is significantly higher than that of the hMSC-laden hydrogels cultured in SCM. 

Concerning the comparison of hMSC-laden MeHA and CS-MeHA hydrogels cultured in SCM, as can be seen in Figure 6, the expression of ACAN increases significantly from day 7 to day 14 of culture for both MeHA and CS-MeHA hydrogels. However, no significant differences in the expression of ACAN can be observed between the two hydrogel types (Figure 6a). On the other hand, the gene expression of Col2A1 is significantly higher in CS-MeHA hydrogels than that of MeHA hydrogels after 7 and 14 days of culture (Figure 6b). Note that for CS-MeHA hydrogels, the expression of Col2A1 exhibits a significant increase from day 7 to day 14 of culture (Figure 6b). On the other hand, the hydrogel type and culture duration do not significantly affect the gene expression of hypertrophy markers Col1A1, Col10A1, or MMP13 (Figure 6c–e). Finally, the hydrogel type and culture time do not significantly affect the value of the Col2A1/Col1A1 ratio (Figure 6f). 

**Figure 7:**
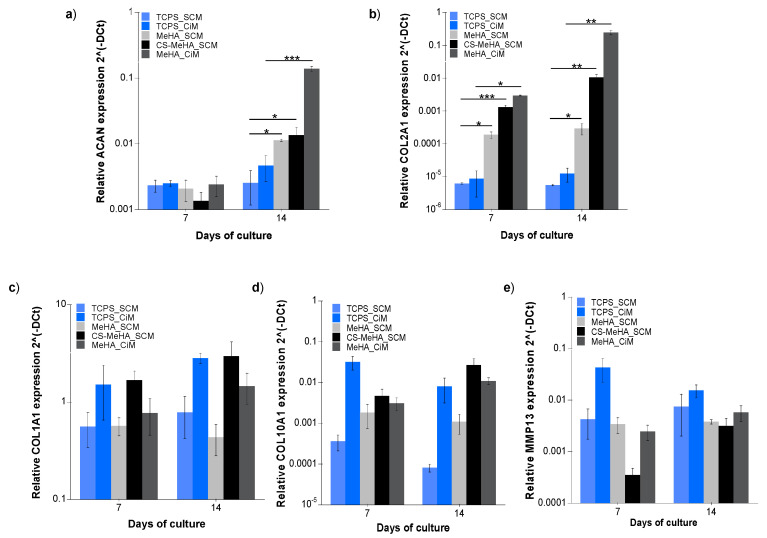
Gene expression analysis of (**a**) ACAN, (**b**) Col2A1, (**c**) Col1A1, (**d**) Col10A1, and (**e**) MMP13 for hMSCs encapsulated in MeHA or CS-MeHA hydrogels, and for hMSCs cultured on tissue culture polystyrene (TCPS) (control) following 7 and 14 days of in vitro culture in SCM or CiM. The data were analyzed with one-way ANOVA and are presented as mean values ± standard error of the mean, * *p* < 0.05, ** *p* < 0.01, *** *p* < 0.001 (*n* = 3 for each donor, hMSCs derived from three different donors).

Figure 7 shows the gene expression of the chondrogenic markers ACAN (5a) and Col2A1 (5b) and the hypertrophic markers Col1A1 (5c), Col10A1 (5d), and MMP13 (5e) after 7 and 14 days of in vitro culture of hMSC-laden MeHA and CS-MeHA hydrogels, as well as of hMSCs on a TCPS (control), in both SCM and CiM media. As can be seen for hMSC-laden MeHA hydrogels cultured for 14 days either in SCM or CiM, the expression of the chondrogenic markers is significantly higher than that of the control culture (i.e., hMSCs cultured on a TCPS). Interestingly, no significant differences can be observed between the expressed hypertrophy markers in the hMSC-laden MeHA hydrogels and those in the hMSCs cultured on a TCPS.

Similarly, it is apparent from the results of Figure 7 that, following 14 days of culture, the expression of the chondrogenic markers in hMSC-laden CS-MeHA hydrogels is significantly higher than that of hMSCs cultured on a TCPS. Moreover, as in the case of hMSC-laden MeHA hydrogels, no significant differences can be observed in the expression of the hypertrophy markers in the hMSC-laden CS-MeHA hydrogels and hMSCs cultured on a TCPS.

### 3.3. Ex Vivo Assessment of Chondrocyte-Laden MeHA Hydrogels

#### 3.3.1. Metabolic Activity of Chondrocytes

**Figure 8:**
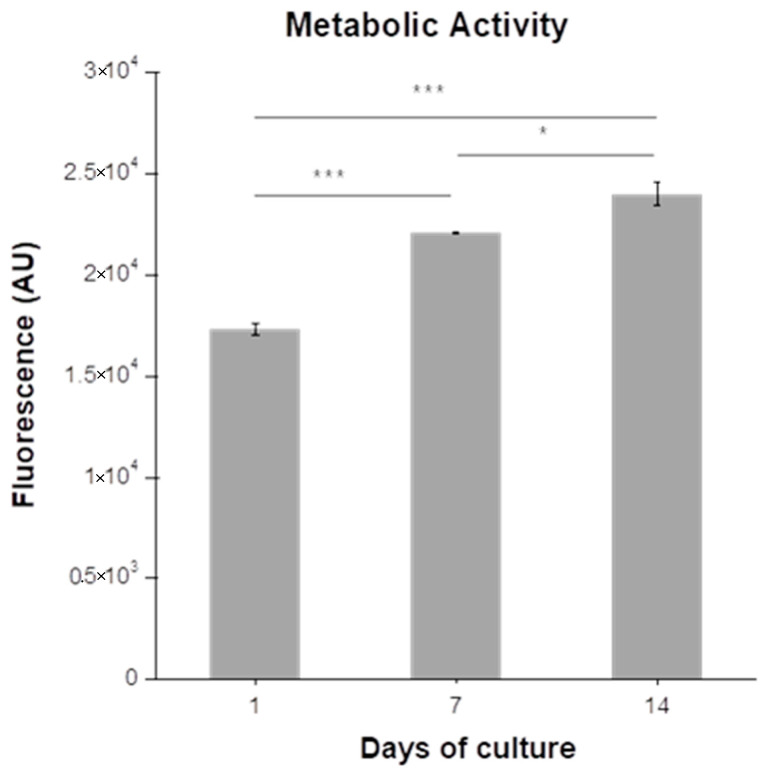
Metabolic activity of chondrocytes encapsulated in MeHA hydrogels (7.5 w/v%). The data were analyzed using one-way analysis of variance (ANOVA) with Tukey’s post hoc test and are presented as mean values ± standard error of the mean (plugs = 6), * *p* < 0.05, ** *p* < 0.01, *** *p* < 0.001.

Figure 8 shows the measured change in the metabolic activity of chondrocytes encapsulated in MeHA hydrogels (7.5 w/v%) during a 14-day culture period. As can be seen, the metabolic activity of the chondrocytes increases significantly throughout the culture period. 

#### 3.3.2. Histological Analysis

**Figure 9:**
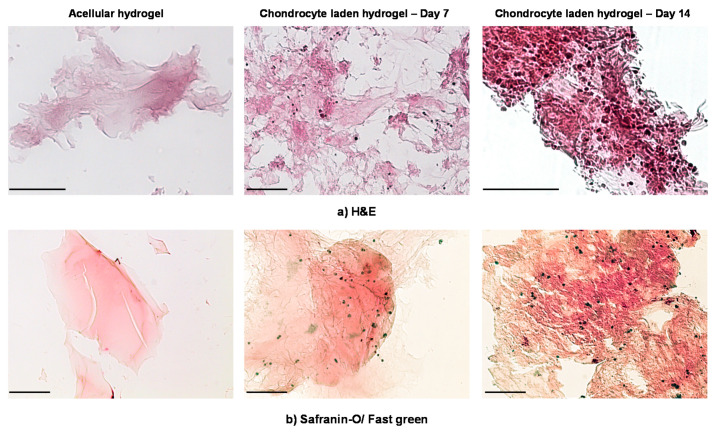
Histological analysis with (**a**) hematoxylin & eosin (H&E) staining and (**b**) Safranin O/Fast Green staining for chondrocyte-laden MeHA hydrogels (7.5 w/v%) extracted from the osteochondral platform after 7 and 14 days of culture. The scale bar corresponds to 100 μm.

In Figure 9a,b, the H&E (Figure 9a) and Safranin O/Fast Green (Figure 9b) staining of the chondrocyte-laden MeHA hydrogels, extracted from the ex vivo osteochondral platform, are depicted at the end of culture days 7 and 14. The H&E and Safranin O/Fast Green stainings of control acellular hydrogels are also shown in Figure 9a,b. As can be seen in Figure 9a, the chondrocytes are homogeneously distributed in the hydrogel matrix. Moreover, the number of chondrocytes exhibits a significant increase from day 7 to day 14 of culture. Note that, at the end of day 14, the chondrocytes appear to form some cell clusters arranged in isogenous groups, characteristic of hyaline cartilage morphology (Figure 9a) [45]. From Figure 9b, one can observe a more intense staining of the chondrocyte-laden MeHA hydrogels on day 14 of culture in comparison to that observed on day 7, indicating a slightly increased accumulation of GAGs (Figure 9b) [46]. Finally, it should be noted that in both cases (i.e., H&E and Safranin O/Fast Green), a slight background staining can be observed, attributed to the MeHA hydrogels.

## 4. Discussion

The developed MeHA and CS-MeHA hydrogels featuring MMP7-cleavable sites were found to exhibit a gelation onset time (e.g., ~500 s) (Table 2, Figure 4a) that permits topical administration via the injection of the macromer/crosslinker solutions and the in situ formation of the hydrogel within the cartilage lesion. CS-MeHA hydrogels were characterized by a lower stiffness (e.g., 2.5 kPa) compared with MeHA hydrogels (e.g., 8.5 kPa) (Table 2) due to the lower degree of crosslinking caused by the restricted accessibility of double bonds (i.e., crosslinkable sites) in the presence of CS, which lowers chain mobility and increases steric hindrance effects. The difference in the rigidity between the two hydrogel types was also confirmed by the experimental results of hydrogel swelling and degradation in the presence of exogenous MMP7 (i.e., recombinant human MMP7). As expected, the softer CS-MeHA hydrogel exhibited a higher degree of swelling and degradation rate compared to the more rigid MeHA hydrogel (Figure 4b,c) due to the lower degree of crosslinking of CS-MeHA polymer chains (i.e., the presence of CS pending peptides). It should be noted that, to date, the optimal swelling ratio that could ensure the best clinical results has not been defined [47]. Regarding hydrogel degradation, since the thiol-methacrylate ester bond is not prone to hydrolysis [48], MeHA and CS-MeHA hydrogel degradation by the recombinant MMP7 enzyme was attributed to the cleavage of the MMP7-sensitive peptide crosslinker [15,19]. Note that high hydrogel rigidity values (e.g., >8 kPa) favor the differentiation of hMSCs into chondrocytes [15,49]. However, various research groups have demonstrated that softer hydrogels can also enhance chondrogenesis and the formation of hyaline cartilage [46,50].

Both MeHA and CS-MeHA hydrogels (5 w/v%) were embedded with bone marrow-derived hMSCs and cultured for 7 and 14 days in SCM. The increased viability of hMSCs could be observed on both culture days, indicating that the hydrogel environment enhances the growth and proliferation of hMSCs (Figure 5a). This observation was also confirmed by the measured DNA content in the hMSC-laden MeHA hydrogels (Figure 5b), which corresponds to the number of viable encapsulated cells. With respect to cell morphology, following 14 days of culture in CiM, the differentiated hMSCs exhibited a chondrocyte-like, round shape, whereas the hMSCs cultured in SCM had a more spread morphology, in agreement with the observations of Feng et al. (2014). In both cases, the morphology of the cells cultured in MeHA and CS-MeHA hydrogels was similar to that of the cells cultured on a TCPS.

The hMSC-laden MeHA hydrogels were cultured for 14 days in either CiM or SCM to assess the effects of culture medium and hydrogel environment on hMSC differentiation. It was found that MeHA hydrogels enhanced the chondrogenic differentiation of hMSCs, especially in the presence of CiM (Figure 6). The expression of the chondrogenic markers in hMSC-laden MeHA hydrogels, cultured in SCM, was found to be lower than that in hMSC-laden MeHA hydrogels cultured in CiM (Figure 6) but significantly higher than that of hMSCs cultured on a TCPS (Figure 7). The latter observation reveals the significant contribution of the hydrogel environment to the differentiation of the hMSCs towards a chondrogenic phenotype. This could potentially be attributed to hMSC–MeHA hydrogel interactions via cell surface receptors [21,51]. The observed ability of the developed MeHA hydrogels to promote the chondrogenesis of the encapsulated hMSCs, even when cultured in an expansion medium, is in agreement with Chung et al., who stated that a scaffold with the appropriate properties is adequate to promote chondrogenesis even in the absence of an induction medium [21]. At this point, it should be noted that only a small number of hMSCs were encapsulated in the hydrogels (e.g., 50,000 cells per hydrogel), compared with other published studies [14,15,46].

Additionally, hMSC-laden CS-MeHA hydrogels were cultured for 14 days in SCM, in order to examine whether the biofunctionalization of MeHA could further promote the differentiation of hMSCs towards a chondrogenic phenotype. The expression of the chondrogenic markers of hMSC-laden CS-MeHA hydrogels cultured in SCM was found to be significantly higher compared to hMSC culture on a TCPS (Figure 7), as well as higher compared to hMSC-laden MeHA hydrogels cultured in SCM (Figure 6), indicating a positive effect of the glycosaminoglycan-binding peptide on the chondrogenic differentiation of hMSCs. On the other hand, the expression of the chondrogenic markers Col2A1 and ACAN of the hMSC-laden CS-MeHA hydrogels cultured in SCM was found to be significantly lower compared to hMSC-laden MeHA hydrogels cultured in CiM (Figure 6). This observation indicates that, although the hydrogel material (MeHA and CS-MeHA) promotes chondrogenesis even in an expansion medium, a chondrogenic medium is still necessary to achieve full-span chondrogenesis [50]. 

Concerning the expression of hypertrophy markers (Col1A1, Col10A1 and MMP13), no significant differences could be observed between hMSC-laden MeHA and CS-MeHA hydrogels cultured in SCM or CiM, and hMSCs cultured on a TCPS (Figure 7). This observation indicated that both MeHA and CS-MeHA hydrogels do not promote the differentiation of hMSCs towards a hypertrophic phenotype, in agreement with Feng et al. (2014).

Finally, MeHA hydrogels were embedded with chondrocytes that were extracted from pig joints. The scope of this study was to examine the ability of chondrocyte-laden MeHA hydrogels to form ECM in an ex vivo cartilage model. The utilization of an ex vivo model gives a better estimation of the regenerative ability of the examined material, since it is possible to assess the hydrogel properties in an environment that mimics native cartilage [43,44,52]. The chondrocytes were directly removed from the same cartilage used to extract the explants for the osteochondral platform and were embedded in the MeHA hydrogels (7.5 w/v% in solids). The reason for selecting a higher solids concentration was the observed higher degradation rate of lower solids MeHA hydrogels (e.g., 5 w/v%) loaded with chondrocytes (data not shown). It should be noted that chondrocytes secrete matrix metalloproteinases during their culture, which degrade the hydrogel matrix by the cleavage of their MMP-degradable sites [20]. A recent proteomic analysis of the chondrocyte secretome has shown MMPs, and specifically MMP1 and MMP3, to be the most abundant secreted enzymes [53]. On the other hand, fully differentiated chondrocytes are not known to secrete MMP7 [15,19,26]. Based on the above considerations and the fact that the MMP7-degradable peptide crosslinker used in this study can be degraded by other MMPs (e.g., MMP1, MMP2) [15], it can be hypothesized that the chondrocyte-laden MeHA hydrogels were degraded by MMPs secreted by the chondrocytes. 

The histological analysis of the chondrocyte-laden MeHA hydrogels, following their extraction from the ex vivo osteochondral platform, revealed a homogeneous distribution of chondrocytes in the hydrogel and an increase in their number from day 7 to day 14 of culture (Figure 9a). This observation was found to be in agreement with the measured metabolic activity of the encapsulated chondrocytes, which also increased significantly between days 7 and 14 of culture (Figure 8), potentially due to an increase in the number of chondrocytes [43]. Additionally, on day 14 of culture, the H&E staining indicated the presence of cell clusters arranged in isogenous groups (Figure 9a), which is a typical morphological characteristic of hyaline cartilage [45]. Finally, the characteristic orange-red color of Safranin O observed on day 14 (Figure 9b), corresponding to GAG deposition, indicated the beginning of ECM formation. It should be noted that these encouraging results were observed despite the low number of encapsulated chondrocytes and the short culture period. 

## 5. Conclusions

The developed MeHA and CS-MeHA hydrogels were found to create a proper environment for the growth and proliferation of hMSCs and to promote their differentiation towards a chondrogenic phenotype, even when cultured in an expansion medium (SCM). This was more pronounced in the case of CS-MeHA hydrogels, indicating the positive effect of the CS-binding peptide on the expression of chondrogenic markers. The histological analysis of chondrocyte-laden MeHA hydrogels cultured in an ex vivo osteochondral platform revealed the formation of cell clusters arranged in isogenous groups, characteristic of hyaline cartilage morphology, and the accumulation of GAGs. These preliminary findings show that the administration of synthesized cell-laden MeHA-based hydrogels, via topical injection, could be considered for the healing of cartilage lesions. However, further studies, including the ex vivo and in vivo evaluation of hMSC-laden MeHA and CS-MeHA hydrogels for longer culture periods and with various cell seeding densities, are needed in order to come to a definite conclusion regarding the performance of the developed hydrogels as cell carriers for the effective treatment of cartilage lesions.

## Figures and Tables

**Table 1:** Sequences of primers used for real-time PCR.

Gene	Forward Primer	Reverse Primer	Base Pairs
*GAPDH*	*5′-GAAGGTGAAGGTCGGAGTCA-3′*	*5′-AATGAAGGGGTCATTGATGG-3′*	108
*COL1A1*	*5′-AGGACAAGAGGCATGTCTGGTT-3′*	*5′-GGACATCAGGCGCAGGAA-3′*	122
*COL2A1*	*5′-CATCCCACCCTCTCACAGTT-3′*	*5′-GCCTCTGCCTTGACCCGAAG-3′*	136
*ACAN*	*5′-TCGAGGACAGCGAGGCC-3′*	*5′-TCGAGGGTGTAGCGTGTAGAGA-3′*	85
*MMP13*	*5′-ACCGGCAAAAGCCACTTTAT-3′*	*5′-TGTCTGGCGTTTTTGGATGT-3′*	109
*COL10A1*	*5′-ACAGGCATAAAAGGCCCACT-3′*	*5′-GACCAGGAGTACCTTGCTCTC-3′*	106

*GAPDH:* glyceraldehyde 3-phosphate dehydrogenase (housekeeping gene) (NM_002046.7); *COL1A1*: collagen type I (NM_000088.4); *COL2A1:* collagen type II (NM_033150.3); *ACAN*: aggrecan (NM_001369268.1); *MMP13*: matrix metalloproteinase 13 (NM_002427.4); *COL10A1*: collagen type X (NM_000493.3).

**Table 2:** Gelation onset time and storage modulus (G’) of methacrylated hyaluronic acid (MeHA) and chondroitin sulfate-methacrylated hyaluronic acid (CS-MeHA) hydrogels (5 w/v%) formed using a matrix metalloproteinase 7 (MMP7)-degradable peptide as a crosslinker in the presence of DMEM. Results are presented as mean values of three measurements ± the respective standard error.

Hydrogel Type	Gelation Onset (s)	G’ (kPa)
MeHA	457 ± 68.1	8.6 ± 1.5
CS-MeHA	529 ± 36.5	2.5 ± 0.2
